# Edible Fungi: Processing, Storage Preservation, Disease Control, and Potential Bioactivities

**DOI:** 10.3390/foods14213755

**Published:** 2025-11-01

**Authors:** Demei Meng, Fansheng Cheng

**Affiliations:** 1 College of Food Science and Engineering, Tianjin University of Science & Technology, Tianjin 300457, China; 2 College of Food Science and Engineering, Qingdao Agricultural University, Qingdao 266109, China

Edible mushrooms have long been recognized for their nutritional and health benefits. However, their high moisture content and active metabolism render them highly perishable post-harvest, resulting in accelerated quality deterioration, including cap browning, stipe elongation, texture softening, and microbial spoilage [1]. These issues pose significant challenges for storage, transportation, and marketability, resulting in substantial economic losses [2]. Consequently, the development of effective preservation and disease control strategies is of the utmost importance for the mushroom industry.

Concurrently, mounting scientific evidence highlights the presence of diverse bioactive compounds in mushrooms, including polysaccharides, terpenoids, and phenolic compounds, which contribute to health-promoting properties such as antioxidant, immunomodulatory, and anti-cancer effects [3,4]. The utilization of these compounds in the development of functional foods, nutraceuticals, and even pharmaceuticals constitutes a pivotal strategy for enhancing the economic value of mushroom industry [5].

This editorial synthesizes recent advances and outlines future perspectives for addressing the challenges and opportunities in the edible mushroom sector, emphasizing sustainable practices and value addition.

This Special Issue, entitled “Edible Fungi: Processing, Storage Preservation, Disease Control, and Potential Bioactivities,” successfully showcases the latest research advances in the field. The eight research papers included in this issue explore key challenges and innovative solutions in the edible mushroom industry from multiple perspectives.

In the area of bioactive component research, Adamczyk et al. conducted an in-depth structural characterization of (1→3)-α-D-glucan from Pleurotus djamor, revealing that this polysaccharide with a molecular weight of 552 kDa contains 86.4% (1→3) linked glucosyl units, laying a theoretical foundation for developing new functional materials.

In the field of nutritional enhancement applications, Hsu et al., through a human clinical trial, confirmed that pulsed UV-treated Pleurotus citrinopileatus significantly increased serum vitamin D_2_ levels. The high-dose group (100 g/day) showed a more than 10-fold increase in vitamin D_2_ levels, while concomitantly reducing parathyroid hormone levels by 37.6%, providing an effective mushroom-based solution for addressing vitamin D deficiency.

Regarding disease control technologies, Lei Zhang et al. found that carvacrol effectively inhibits Pseudomonas tolaasii. Its mechanism of action includes the disruption of bacterial cell membranes, the activation of the mushroom’s defense system, and the promotion of the accumulation of antimicrobial substances. The efficacy of low-concentration fumigation at 20 μmol/L in the management of brown blotch disease was demonstrated.

In the area of preservation technology optimization, Yuxian Yang et al. demonstrated that controlled atmosphere storage with 1–3% O_2_ and 15–17% CO_2_ effectively delayed browning in Agaricus bisporus. In a separate study, Yalong Guo et al. optimized the best parameters for cold plasma treatment (95 kV, 130 Hz, 10 min) using response surface methodology, significantly extending mushroom shelf life.

In the field of pathogen genomics, Yufei Lan et al. made a significant contribution by completing the genome sequencing of three cobweb disease pathogenic fungi for the first time, revealing characteristics of their pathogenicity-related genes, providing a molecular basis for disease control.

In terms of processing and utilization innovation, Wenliang Wang et al. discovered that the incorporation of 10% Flammulina velutipes soluble dietary fiber improved noodle quality. Concurrently, the review by Akruti Singh et al. systematically summarized the bioactive components and health benefits of 11 edible mushroom species.

These research findings provide important theoretical support and technical foundations for the sustainable development of the edible mushroom industry, thereby promoting the transition from fundamental research to its practical application in industry. Future research will persist in concentrating on green control, precise preservation, and high-value utilization, thereby further unleashing the development potential of the edible mushroom industry.

**Prospective Research Directions**

Despite significant progress, future research should focus on several key areas to overcome existing challenges and fully exploit the potential of edible mushrooms:

**Green and Safe Disease Control Technologies**: Developing strategies based on plant essential oils (e.g., thymol), antagonistic microorganisms, and induced resistance to reduce reliance on chemical pesticides, ensuring product safety and environmental sustainability.

**Integrated Preservation Methods**: Combining physical techniques (e.g., cold plasma, pulsed light, and edible coatings), biological control, and smart packaging to establish a full-chain quality assurance system from postharvest to consumption.

**Efficient Comprehensive Utilization of Bioactive Compounds**: Applying advanced extraction technologies (e.g., ultrasound-assisted, microwave-assisted, enzymatic, and supercritical fluid extraction) to obtain bioactive compounds. Research should focus on elucidating their structure–activity relationships and developing high-value products, such as functional foods, nutraceuticals, and cosmeceuticals, to address product homogenization and drive industry upgrading.

The mushroom industry is evolving into an independent agricultural system, promising to form a tripartite agricultural (plant, animal, and mushroom agriculture). As a high-quality protein source with an amino acid profile comparable to animal proteins, mushrooms can alleviate protein supply pressures, particularly in developing countries [4]. Market trends, such as a 223-fold consumption increase in Japan over two decades, reflect growing consumer awareness and a solid industrial foundation. Supported by favorable policies, the utilization of abundant agricultural waste, and easily transferable cultivation techniques, the mushroom industry is poised to become a powerhouse for rural economic development worldwide. It creates new income streams, diversifies agricultural production, and provides new momentum for sustainable agricultural transformation, particularly in developing regions.
